# Prevalence of *Mollicutes* among men who have sex with men and transgender women aged 15 to 19 years in Salvador, North-eastern Brazil

**DOI:** 10.1186/s12879-023-08213-z

**Published:** 2023-04-18

**Authors:** Valdiele de Jesus Salgado, Caio Marcellus Pereira de Abreu Oliveira, Ágatha Morgana Bertoti da Silva, Henrique Inácio Lima de Brito, Danielle Souto de Medeiros, Fabiane Soares, Laio Magno, Inês Dourado, Guilherme Barreto Campos, Lucas Miranda Marques

**Affiliations:** 1 State University of Santa Cruz, Rod. Jorge Amado, Km 16, Salobrinho, Ilhéus, Bahia 45662-900 Brazil; 2 Multidisciplinary Health Institute, Federal University of Bahia, Rua Hormindo Barros, 58, Candeias, Vitória da Conquista, Bahia 45029-094 Brazil; 3 Institute of Collective Health, Federal University of Bahia, Av. Adhemar de Barros, S/nº, Ondina, Salvador, Bahia 40170-110 Brazil; 4 Department of Life Sciences, State University of Bahia, Rua Silveira Martins, 2555, Salvador, Bahia 41000-150 Brazil

**Keywords:** PrEP, STI, Prevalence, *Mollicutes*, *Mycoplasma*, *Ureaplasma*

## Abstract

**Background:**

Some species of *Mollicutes* have been associated with different pathologies of the urogenital tract in humans, with a high prevalence among adult men who have sex with men (MSM) and transgender women (TGW). However, few studies have been performed to investigate its prevalence among adolescents. In this study, we estimated the initial prevalence of *Mycoplasma genitalium* (MG), *Mycoplasma hominis* (MH), *Ureaplasma urealyticum* (UU), and *Ureaplasma parvum* (UP); the rate of misdiagnosis at different anatomical sites; and the associated factors with positive tests for *Mollicutes* among MSM and TGW aged 15 to 19 years enrolled in the PrEP1519 study.

**Methods:**

PrEP-1519 is the first study to investigate the effectiveness of pre-exposure prophylaxis for human immunodeficiency virus among adolescent MSM and TGW aged 15 to 19 in Latin America. Oral, anal, and urethral swabs were taken from 246 adolescents upon enrolment in the study to detect MG, MH, UU, and UP by quantitative polymerase chain reaction (qPCR). Bivariate and multivariate analyses were conducted by Poisson regression and 95% confidence intervals (95% CI) were estimated.

**Results:**

The prevalence of *Mollicutes* was 32.1%. UU was the most prevalent species (20.7%), followed by MH (13.4%), MG (5.7%), and UP (3.2%); 67.3% of the positive samples would have been missed if only urethral samples had been taken. Receptive anal sex (prevalence ratio [PR] = 1.79; 95% CI = 1.07–3.01) and clinical suspicion of sexually transmitted infection (PR = 1.62; 95% CI = 1.01–2.61) were factors associated with the detection of *Mollicutes* in general. Group sex (PR = 1.98; 95% CI = 1.12–3.50) and receptive anal sex (PR = 2.36; 95% CI = 0.95–5.86) were associated with the detection of *Mycoplasma* spp. No sociodemographic, clinical, or behavioural variable was significantly associated with the detection of *Ureaplasma* spp.

**Conclusions:**

A high prevalence of *Mollicutes* was observed among adolescent MSM and TGW, especially at extragenital sites. Further research is required to understand the epidemiological profile of high-risk adolescents in different regions and contexts, and to investigate the pathogenesis of *Mollicutes* in the oral and anal mucosa before routine screening can be recommended in clinical practice.

**Supplementary Information:**

The online version contains supplementary material available at 10.1186/s12879-023-08213-z.

## Background

Sexually active adolescents constitute a population group that is vulnerable to sexually transmitted infections (STIs) due to several factors of psychosocial and biological nature [1–5]. In addition, adolescents are less likely to be screened for STIs due to anonymity and confidentiality concerns [6]. For some population groups, such as men who have sex with men (MSM) and transgender women (TGW), access to health services is even more challenging, as they are often stigmatized and discriminated in the community and by health workers [7–11], forming barriers to the prevention, treatment, and control of STIs in this population. Furthermore, these barriers are associated with higher rates of human immunodeficiency virus (HIV) infection and other STIs among these populations, regardless of age, thereby also including adolescents and young individuals [12–14]. In addition, MSM tend to have more sexual partners, concurrent partners, and older partners [15–17], along with unprotected anal intercourse [18, 19]. In relation to TGW, a significant proportion of TGW engage in commercial sexual relationships exposing them to work in precarious conditions and experience sexual violence, often rendering them unable to negotiate the use of condoms with their clients, and thereby increasing the risk of HIV and other STIs [20]. Screening these populations is therefore imperative for the diagnosis and treatment of STIs and the promotion of more adequate prevention and care [21, 22].

Data on the prevalence of STIs and associated factors, along with the risk of acquiring STIs, among adolescent MSM (AMSM) and TGW (ATGW) are extremely limited. Regarding the prevalence of *Mollicutes* species, the gap in data availability is even greater, since they rarely cause a symptomatic infection, which is one of the reasons why they are neglected in epidemiological surveillance and research. Despite this, this species has been associated with several pathologies of the urogenital tract, such as non-chlamydial non-gonococcal urethritis [23–25], male infertility [26–28], an increased risk of HIV transmission [29, 30], and antibiotic resistance [31]. This species is highly prevalent in MSM over the age of 18 [32–36], even in extragenital sites (i.e., oral and anal) [37, 38], and is hard to identify in clinical practice due to the absence of symptoms, making them important extragenital reservoirs [37–41]. A missed diagnosis then leads to the persistence of these microorganisms and their potential transmittance to sexual partners [39–42]. Furthermore, the presence of other common STIs (i.e., chlamydia and gonorrhoea) at extragenital sites of AMSM and ATGW [13] suggests that *Mollicutes* may be present at different anatomical sites. The aim of this study was to estimate the prevalence of *Mycoplasma genitalium* (MG), *Mycoplasma hominis* (MH), *Ureaplasma urealyticum* (UU), and *Ureaplasma parvum* (UP) at different anatomical sites to assess the rate of missed detection and to analyse factors associated with the prevalence of *Mollicutes* among AMSM and ATGW.

## Methods

### Study design and population

A cross-sectional study based on baseline data obtained from the PrEP1519 cohort study was conducted in three Brazilian cities – Salvador, Belo Horizonte, and São Paulo – which aimed to analyse the effectiveness of HIV pre-exposure prophylaxis (PrEP) among adolescents from key populations who had a history of risk exposure to or vulnerability to HIV infection (e.g. condomless anal sex in the previous 6 months, a history of STI or use of post-exposure prophylaxis (PEP) to HIV in the previous 12 months, commercial sex work). Recruitment of participants was conducted by demand creation strategies on youth social venues and virtual networks (YouTube, Instagram, TikTok, Whatsapp, Facebook and Twitter), including dating apps (Grindr, Tinder and Badoo). Also, public health services and non-governmental organizations (NGOs) could refer eligible participants to the study.

In the present study, baseline data from April 2019 to February 2021 from the city of Salvador, the capital of the North-eastern Brazilian state of Bahia, was analysed. The inclusion criteria were AMSM and ATGW aged 15–19 years old (y/o), who had at least one act of sexual intercourse with another cisgender man or TGW in the past 12 months and had spent most of their time at the study site (i.e., living, studying, working, or residing in one of the study sites). Participants of the PrEP1519 study were offered PrEP and other combination prevention strategies, sexual health care, and STI testing, including those for bacterial STIs, with quarterly follow-ups conducted for up to 3 years.

After checking the eligibility criteria and the information on the proposed steps for the study, individuals who agreed to participate in the study provided written informed consent or assent and were tested at the initial visit (baseline). Cotton swab samples from oral, urethral, and anal sites for MG, MH, UU, and UP were collected to perform real-time polymerase chain reaction (PCR) tests. A socio-behavioural questionnaire was provided at baseline. This study was approved by the Research Ethics Committee of the World Health Organization (Protocol ID: Fiotec-PrEP Adolescent study) and the Federal University of Bahia (# 3,224,384).

### Data collection

A socio-behavioural questionnaire with questions about gender identity, access to health services, sexual practices, drug and alcohol use, and situations of violence was administered at baseline by trained investigators and was used for data analysis. The sociodemographic variables included: age (< 18 or ≥ 18 y/o), race/colour (non-black [white, brown, yellow, other] or black), schooling (elementary school/adult education or high school/higher education), population group (MSM or TGW), and sexual orientation (homosexual/gay/lesbian or bisexual/heterosexual). Behavioural variables were steady sexual partner in the last 3 months (yes or no), casual sexual partner in the last 3 months (yes or no), receptive anal sex (yes or no), insertive anal sex (yes or no), group sex (yes or no), interference of drugs or alcohol in condom use (yes or never), and condom use in the last 3 months (consistent or inconsistent). The clinical variable was clinical suspicion of STI (normal test result or altered test result), which consisted of signs and symptoms suggestive of STI, such as urethral discharge, presence of warts and lesions, dysuria, itching, and irritation, among others.

### Collection and storage of biological samples

Oropharyngeal, anorectal, and urethral cotton swabs were taken during the consultation with a doctor or nurse upon enrolment in the study. For the oropharyngeal swabs, the tongue was pressed down using a sterile tongue depressor to avoid contact with the tongue, cheeks, palate, and uvula, and the hydrophilic swab was then rubbed on the tonsils and behind the uvula. For the anorectal sample, the participant was asked to lie on their side with one leg slightly flexed. The swab was then introduced one to two centimetres beyond the rectal sphincter and rotated. For the urethral sample, the foreskin was retracted, exposing the glans, and a paediatric swab was carefully inserted about two centimetres into the urethral meatus and rotated. All samples were stored in 5 mL transport medium at 4ºC until processing [43]. In the laboratory, the samples were homogenized by vortexing (30 s), divided into 1 mL aliquots in microtubes, and were then stored in the refrigerator (-20 °C) until they were used.

### Extraction of DNA

The DNA extracted from the samples was obtained using a boiling method with phosphate-buffered saline solution [44]. After extraction, the DNA was quantified and analysed for the presence of contaminants (lipids and proteins) using a NanoDrop™ 2000 spectrophotometer (Thermo Scientific, Brazil) (OD 260/280 and 260/230 ratio).

### Conventional and real-time PCR

PCR tests were performed to screen for samples with *Mollicutes* using primers to amplify a fragment with 280 bp [45], verified by agarose gel electrophoresis and UV photodocumentation. The positive or indeterminate samples were then submitted to a real-time quantitative PCR (qPCR) assay, performed in a StepOnePlus™ Real-Time PCR System (Applied Biosystems, Brazil), using a TaqMan™ probe and TaqMan™ Universal PCR Master Mix (Thermo Fisher Scientific, Waltham, MA, USA) to amplify the target genes of MG, MH [46], UU, and UP [47]. The microbial DNA of each microorganism was obtained from the Mycoplasma Laboratory of the University of São Paulo (Brazil) and used as a positive control. Milli-Q water was used as a negative control for detecting contaminants. Positivity was determined when the signal from the sample crossed the threshold, as calculated by the equipment based upon the cycle threshold (Ct) of the positive control.

### Statistical analyses

The bacterial load of the positive samples was estimated from the cycle threshold value using the StepOne™ system (Applied Biosystems, Brazil). To assess whether there was a difference in the load of the different species and at different anatomical sites, the comparisons made were determined by individual variance or error variance (s^2^), by the Shapiro–Wilks normality test, and the Mann–Whitney U test, because the data distribution was not normal.

To estimate the missed detection of *Mollicutes* when a particular anatomical site was not screened, the following calculation was performed (see equation below), and Fisher’s exact test was used to calculate statistical significance (*p* ≤ 0.05):

$$\frac{\left(\mathrm{\%\;at\;that\;site }-\mathrm{total\;\%}\right)}{\mathrm{total\;\%}}$$

The sociodemographic and behavioural variables were taken as independent variables for the bivariate analysis. The following dependent (outcome) variables were considered: presence (or not) of each species (MG, MH, UU, UP) of the genera (*Mycoplasma* spp. and *Ureaplasma* spp.) or the class (*Mollicutes*) analysed individually. The bivariate analyses were conducted using Poisson regression with robust variance, estimating the prevalence ratio (PR) and respective 95% confidence interval (CI). The differences between the variables and the occurrence of the outcome were tested using Pearson’s chi-squared test or Fisher’s exact test (when at least one of the expected values was under 5), with significance set at 5% (p ≤ 0.05). For the multivariate analysis, backward stepwise regression was used. The adequate model was selected according to the Akaike information criterion and Bayesian information and was then assessed using the chi-squared test. Stata 15.0 (Stata Corporation, College Station, USA) was used for these analyses.

## Results

A total of 246 adolescents were enrolled in the study, most of whom were 18 years or above (85.4%; median age, 18.83 years [18.20–19.44]), were high school or university students (89.3%), were self-identified as black (85.8%), MSM (93.9%), homosexual (64.2%), and had inconsistent condom use (60.7%) (Table 1). Regarding collection refusal, 36 and 33 participants refused the collection of anal and urethral samples, respectively. One oral sample was lost due to mishandling during transportation to the laboratory.

**Table 1:** Sociodemographic and behavioural characteristics of AMSM and ATGW enrolled in the PrEP1519 study (*N* = 246) in Salvador, Brazil, from April 2019 to February 2021

Variables	n	%
**Sociodemographic**		
** Age**		
15–17 yo	36	14.6
18–19 yo	210	85.4
** Race/Colour**		
Not Black	35	14.2
Black	211	85.8
** Schooling**		
Elementary school/Adult education	26	10.7
High school/Higher education	218	89.3
** Population group**		
AMSM	231	93.9
ATGW	15	6.1
** Sexual Orientation**		
Homosexual/Gay/Lesbian	158	64.2
Bisexual/Heterosexual	88	35.8
**Behavioural**		
** Steady sexual partner in the last three months**		
No	111	45.5
Yes	133	54.5
** Casual sexual partner in the last three months**		
No	84	34.4
Yes	160	65.6
** Receptive anal sex**		
No	68	27.9
Yes	176	72.1
** Insertive anal sex**		
No	97	39.7
Yes	147	60.2
** Condom use in the last three months**		
Consistent	96	39.3
Inconsistent	148	60.7
** Group sex**		
No	204	83.6
Yes	40	16.4
** Interference of alcohol use in condom use**		
No	169	80.1
Yes	42	19.9
** Interference of drugs in condom use**		
No	103	87.3
Yes	15	12.7
**Clinical**		
** Clinical suspicion of STI**		
Normal test result	212	90.3
Altered test result	23	9.7

*AMSM* Adolescent men who have sex with men, *ATGW* Adolescent transgender women, *STI* Sexually transmitted infection

The overall prevalence of *Mollicutes*, i.e., the proportion of participants who tested positive for any of the four species investigated at any swab site (oral, anal, urethral) was 32.1% (95% CI: 26.5–38.2). UU was the most prevalent species (20.7%, 95% CI: 16.1–26.3), followed by MH (13.4%, 95% CI: 9.7–18.3), MG (5.7%, 95% CI: 3.4–9.4), and UP (3.2%, 95% CI: 1.6–6.4). The two swab sites with the highest prevalence of infection were oral (19.6%) and anal (19.5%), followed by urethral (14.1%).

A high rate of missed detection at the extragenital sites (i.e., oral and anal) was observed when the prevalence per site was compared with the prevalence at all three sites (Table 2). A significant statistical difference was observed in the detection of UU, with a missed detection of 56.5%, 51.7%, and 66.2% if only the oral, anal, and urethral samples were analysed, respectively. For MH, the missed detection rate was 51.5%, 57.5%, and 68.7% if only the oral, anal, and urethral samples were analysed, respectively. Significant statistically missed detection was also observed for MG for the analysis of samples from the urethra (75.4%), as described in Table 2.

**Table 2:** Prevalence of *Mycoplasma genitalium, Mycoplasma hominis*, *Ureaplasma parvum*, and *Ureaplasma urealyticum* in oral, anal, and urethral swabs, and the missed detection of *Mollicutes* among AMSM and ATGW enrolled in the PrEP1519 study in Salvador, Brazil (*N* = 246), April 2019 to February 2021

Species	Three Swab Sites (*N* = 246) n (%)	Oral (*N* = 245) n (%)	Missed Detection Rate (%)	*p*-value	Anal (*N* = 210) n (%)	Missed Detection Rate (%)		*p*-value	Urethral (*N* = 213) n (%)	Missed Detection Rate (%)	*p*-value
**MG**	14 (5.7)	8 (3.3)	42.1	0.275	5 (2.9)	49.1	0.100		3 (1.4)	75.4	**0.023**
**MH**	**33 (13.4)**	16 (6.5)	51.5	**0.015**	12 (5.7)	57.5	**0.007**		9 (4.2)	68.7	**0.001**
**UU**	**51 (20.7)**	22 (9.0)	56.5	**0.001**	21 (10.0)	51.7	**0.002**		15 (7.0)	66.2	**0.000**
**UP**	8 (3.2)	2 (0.8)	75.0	0.106	3 (1.4)	56.2	0.237		3 (1.4)	56.2	0.234
**All**	79 (32.1)	48 (19.6)	54.5	** < 0.001**	41 (19.5)	54.7	** < 0.001**		30 (14.1)	67.3	** < 0.001**

Legend: *MG M. genitalium*, *MH M. hominis*, *UP U. parvum*, *UU U. urealyticum*, *AMSM* adolescent men who have sex with men, *ATGW* adolescent transgender women. *N* = 246 (absolute number of participants). Figures in bold indicate the highest prevalence and significantly different missed detection rates

Supplementary Figure S1 shows the Ct value of the positive samples for each species of *Mollicutes* at each swab site. Statistically significant differences were found for MG (*p* = 0.0008) and UU (*p* = 0.0087), which had lower Ct values at the anal and urethral sites, respectively, meaning that there was a greater bacterial load of MG at the anal site and a greater bacterial load of UU at the urethral site, thus, a greater risk of infection.

Of the total confirmed *Mollicutes* infections, 69.6% (55/79) were mono-infections, with 10.9% (6/55) of infections by the same microorganism at more than one anatomical site. As for others, 31.6% (25/79) were co-infections (simultaneous infections. with two or more microorganisms), of which UU and MH were the most prevalent (Table 3).

**Table 3:** Prevalence of co-infections amongst AMSM and ATGW enrolled in the PrEP1519 study in Salvador, Brazil, from April 2019 to February 2021

Microorganisms	Total (*N* = 246) n (%)	AMSM	ATGW
		**(*n* = 231)**	**(*n* = 15)**
		**n (%)**	**n (%)**
MH + UU	**16 (6.5)**	**14 (6.9)**	**2 (13.3)**
MG + UU	3 (1.2)	3 (1.3)	-
MG + MH	1 (0.4)	1 (0.4)	-
MH + UP	1 (0.4)	1 (0.4)	-
UP + UU	1 (0.4)	1 (0.4)	-
MG + MH + UU	3 (1.2)	3 (1.3)	-
Total no. of participants with co-infections	25 (10.2)	23 (9.9)	2 (13.3)

Legend: *MG Mycoplasma genitalium*, *MH Mycoplasma hominis*, *UP Ureaplasma parvum*, *UU Ureaplasma urealyticum*. *AMSM* adolescent men who have sex with men, *ATGW* adolescent transgender women. *N* = total number of participants screened. Totals in bold indicate the highest prevalence (over 5%)

In the bivariate analysis, having had receptive anal sex (PR = 2.62, 95% CI = 1.07–6.42) and having had group sex (PR = 2.53, 95% CI = 1.42–4.51) were associated with the detection of *Mycoplasma* spp. None of the variables investigated were significantly related to the detection of *Ureaplasma* spp. Meanwhile, the only factor that was positively associated with the detection of *Mollicutes* was receptive anal sex (PR = 1.74, 95% CI = 1.02–2.95) (Table 4). As for the species, only group sex was associated with detecting MH (PR = 2.56, 95% CI = 1.29–5.09). The other variables were not associated with any particular species or had very large confidence intervals, for the analyses of UP, compromising the validity of the results (Supplementary Table 1).

**Table 4:** Bivariate analysis of the prevalence of *Mycoplasma* spp., *Ureaplasma* spp., and *Mollicutes* among AMSM and TGW (*N* = 246); PrEP1519, Salvador, Brazil, April 2019 to February 2021

**Variables**	*Mycoplasma* spp.					*Ureaplasma* spp.					*Mollicutes*				
	n*	**P(%)**^†^	**PR**^‡^	**95% CI**^§^	***p*-value**	n*	**P(%)**^†^	**PR**^‡^	**95% CI**^§^	***p*-value**	n*	**P(%)**^†^	**PR**^‡^	**95% CI**^§^	***p*-value**
**Age**															
15–17 yo	6	18.8	1.00	-	-	11	34.4	1.00	-	-	12	37.5	1.00	-	-
18–19 yo	34	17.1	1.10	0.50–2.41	0.816	41	20.6	0.67	0.96–2.90	0.069	60	30.2	0.86	0.76–2.04	0.389
**Race/Colour**															
Not Black	4	12.1	1.00	-	-	6	18.2	1.00	-	-	8	24.2	1.00	-	-
Black	36	18.2	1.50	0.57–3.95	0.411	46	23.2	1.28	0.59–2.76	0.532	64	32.3	1.33	0.70–2.52	0.376
**Schooling**															
Elementary school/Adult education	1	5.6	1.00	-	-	3	16.7	1.00	-	-	4	22.2	1.00	-	-
High school/Higher education	37	17.5	3.16	0.46–21.77	0.243	48	22.8	1.36	0.47–3.96	0.567	66	31.3	1.41	0.58–3.42	0.451
**Sexual Orientation**															
Homosexual/Gay/Lesbian	28	17.9	1.00	-	-	37	23.7	1.00	-	-	51	32.7	1.00	-	-
Bisexual/Heterosexual	12	16.0	0.89	0.48–1.66	0.716	15	20.0	0.84	0.49–1.44	0.532	21	28.0	0.86	0.56–1.31	0.478
**Steady sexual partner in the last three months**															
No	14	13.7	1.00	-	-	27	26.5	1.00	-	-	29	28.4	1.00	-	-
Yes	24	18.9	1.38	0.75–2.53	0.302	24	18.9	0.71	0.44–1.16	0.174	41	32.3	1.14	0.76–1.69	0.532
**Casual sexual partner in the last three months**															
No	11	13.9	1.00	-	-	18	22.8	1.00	-	-	25	31.7	1.00	-	-
Yes	27	18.0	1.29	0.68–2.47	0.437	33	22.0	0.97	0.58–1.60	0.892	45	30.0	0.95	0.63–1.42	0.797
**Receptive anal sex**															
No	5	7.7	1.00	-	-	13	20.0	1.00	-	-	13	20.0	1.00	-	-
Yes	33	20.1	**2.62**	**1.07–6.42**	**0.036**	38	23.2	1.16	0.66–2.03	0.608	57	34.8	**1.74**	**1.02–2.95**	**0.041**
**Insertive anal sex**															
No	10	11.9	1.00	-	-	17	20.2	1.00	-	-	22	26.2	1.00	-	-
Yes	28	19.3	1.62	0.83–3.17	0.158	34	23.5	1.16	0.69–1.94	0.577	48	33.1	1.26	0.82–1.94	0.284
**Condom use in the last three months**															
Consistent	11	12.2	1.00	-	-	20	22.2	1.00	-	-	25	27.8	1.00	-	-
Inconsistent	27	19.4	1.59	0.83–3.05	0.163	31	22.3	1.00	0.61–1.65	0.989	45	32.4	1.17	0.77–1.76	0.466
**Group sex**															
No	25	13.2	1.00	-	-	43	22.6	1.00	-	-	55	28.9	1.00	-	-
Yes	13	33.3	**2.53**	**1.42–4.51**	**0.002**	8	20.5	0.91	0.46–1.78	0.775	15	38.5	1.33	0.84–2.09	0.222
**Interference of alcohol in condom use**															
No	28	17.6	1.00	-	-	35	22.0	1.00	-	-	48	30.2	1.00	-	-
Yes	8	20.5	1.12	0.38–3.31	0.842	10	25.6	1.16	0.63–2.15	0.624	15	38.5	1.27	0.80–2.02	0.305
**Interference of drugs in condom use**															
No	19	19.2	1.00	-	-	23	23.2	1.00	-	-	33	33.3	1.00	-	-
Yes	3	21.4	0.98	0.33–2.91	0.972	2	14.3	0.61	0.16–2.34	0.476	4	28.6	0.86	0.36–2.06	0.731
**Clinical suspicion of STI**															
Normal test result	33	16.6	1.00	-	-	44	22.1	1.00	-	-	60	30.2	1.00	-	-
Altered test result	6	28.6	1.72	0.82–3.63	0.153	6	28.6	1.29	0.62–2.67	0.489	9	42.9	1.42	0.83–2.44	0.201

AMSM = adolescent men who have sex with men, TGW = transgender women; STI, sexually transmitted infection

^*^n = absolute frequency of detection of the microorganism;

^†^*P* = prevalence of the microorganism;

^‡^PR = crude prevalence ratio;

^§^95% CI: 95% confidence interval

Statistically significant variables (*p* < 0.05) marked in bold

In the final multivariate analysis model, factors associated with positivity of *Mollicutes* were receptive anal sex (PR = 1.78, 95% CI = 1.03–3.09) and clinical suspicion of STI (PR = 1.46, 95% CI = 1.01–2.49). Having had group sex (PR = 1.97, 95% CI = 1.08–3.59) was associated with testing positive for *Mycoplasma* spp. No sociodemographic, behavioural, or clinical variable was significantly associated with *Ureaplasma* spp. As for the species, having had group sex (PR = 2.19, 95% CI = 1.09–4.38) was associated with the detection of MH. No variable was statistically significantly associated with testing positive for MG and UU. As for UP, its low prevalence in our study population (3.2%) indicated that the confidence intervals of the variables were very large, compromising the validity of the results (Table 5).

**Table 5:** Final Poisson regression model for the selected groups of variables for *Mollicutes*, *Mycoplasma* spp., and *Ureaplasma* spp. among adolescents (*N* = 246); PrEP1519, Salvador, Brazil, April 2019 to February 2021

Positive test for *Mollicutes*				
**Variables**	PR^*^	**95% CI**^†^		***p*-value**
**Receptive anal sex**				
No	1.00	-		-
Yes	**1.78**	**1.03–3.09**		**0.039**
**Clinical suspicion of STI**				
Normal test result	1.00	-		-
Altered test result	**1.46**	**1.01–2.49**		**0.041**
**Positive test for *Mycoplasma* spp.**				
**Variables**	PR^*^	**95% CI**^†^		***p*-value**
**Receptive anal sex**				
No	1.00	-		-
Yes	2.10	0.85–5.23		0.109
**Group sex**				
No	1.00	-		-
Yes	**1.97**	**1.08–3.59**		**0.027**
**Clinical suspicion of STI**				
Normal test result	1.00	-		-
Altered test result	1.60	0.77–3.35		0.208
**Positive test for *Ureaplasma* spp.**				
**Variables**	PR^*^	**95% CI**^†^		***p*-value**
**Age**				
15–17 yo	1.00	-		-
18–19 yo	0.68	0.99–2.85		0.056
**Steady sexual partner in the last three months**				
No	1.00	-		-
Yes	0.72	0.45–1.16		0.180
**Positive test for *M. genitalium***				
**Variable**	PR^*^	**95% CI**^†^		***p*-value**
**Condom use in the last three months**				
Consistent	1.00	-		-
Inconsistent	3.31	0.71–15.31		0.126
**Group sex**				
No	1.00	-		-
Yes	1.82	0.58–6.02		0.299
**Positive test for *M. hominis***				
**Variables**	PR^*^	**95% CI**^†^		***p*-value**
**Receptive anal sex**				
No	1.00	-		-
Yes	2.02	0.72–5.72		0.183
**Group sex**				
No	1.00	-	-	
Yes	**2.19**	**1.09–4.38**	**0.027**	
**Positive test for *U. urealyticum***				
**Variables**	PR^*^	**95% CI**^†^	***p*-value**	
**Sexual Orientation**				
Homosexual/Gay/Lesbian	1.00	-	-	
Bisexual/Heterosexual	0.63	0.32–1.24	0.181	
**Interference of alcohol in condom use**				
No	1.00	-	-	
Yes	1.33	0.72–2.49	0.365	

*PR* crude prevalence ratio, *CI* confidence interval, *STI* sexually transmitted infection, *MSM* men who have sex with men, *TGW* transgender women

## Discussion

This is the first study to analyse the prevalence of *Mollicutes* at three anatomical sites in 15 to 19-year-old MSM and TGW in a Brazilian city. The two most prevalent species in the study population were UU and MH, with a prevalence of 20.7% and 13.4%, respectively, which can be considered high. Although population group, age group, and samples collected differed, a study by Park et al. (2017) [48] that analysed urine samples obtained from 12 to 19-year-old heterosexual adolescents also found a high prevalence of these species (27.4% for UU and 17.3% for MH). Another study that included MSM aged 18 and over found a high prevalence (around 24.3%) of both species in the samples from anal swabs [38].

UU is possibly an etiological agent of non-gonococcal urethritis [49, 50], with a prevalence of 5% to 26% in acute urethritis [51], and is also associated with male infertility [26–28]. MH presents intracellular parasitic behaviour in gametic cells, altering semen parameters such as sperm count, motility, and morphology, with a higher prevalence found in infertile (14.5%) than fertile (3.6%) men, which suggests it could also play a role in male infertility [52]. Even so, screening for these species and for UP in asymptomatic, and even symptomatic, individuals are not yet recommended since most of the patients carrying them do not develop the disease. Antimicrobial treatment for eradicating this microorganism is complex and is not clearly associated with a cure; it could even select or induce microbial resistance in mycoplasmas and more severe STIs [53].

The prevalence of MG found in our study was 5.7% and we considered this to be high. It is similar to the prevalence of 5.1% and 6.6% found by Reinton et al. (2013) [37] and Soni et al. (2010) [42], respectively, in anal and oral swabs and urine samples. In a study by Park et al. (2017) [48], the prevalence in the urine samples taken from heterosexual adolescents aged 12 to 19 was 4.2% and was also low as compared to UU and MH.

Of all the *Mollicutes* species studied, MG is regarded as an emerging STI in many countries, especially in Europe, where it contributes to 10% to 35% of non-chlamydial non-gonococcal urethritis in men [54]. Further, there is evidence that MG is an important etiological agent of proctitis in MSM [55] and that inflammation may facilitate the transmission and acquisition of HIV [33, 42]. In this context, screening and adequate treatment for anorectal MG infections is also a strategy for the prevention of HIV, especially for individuals with high-risk sexual behaviours, such as anal sex without the use of a condom [42]. As such, unlike other species, guidelines for its screening and treatment have already been established [21, 56], advising men with recurring non-gonococcal urethritis and women with recurring cervicitis to get tested for MG, preferably with simultaneous genotypic resistance testing to enable the best choice of antibiotic therapy [57], since some strains of MG have presented clear evidence of antimicrobial resistance to macrolides (like azithromycin) and resistance to quinolones (like moxifloxacin) [31, 58–61].

One point worth highlighting is treatment failure, which not only induces resistance but can also contribute to the persistence of this pathogen and its transmission to sexual partners during sex without the use of condoms [62]. Therefore, screening for MG and prescribing treatment should be weighed carefully in clinical practice. In addition, increased screening in the population would lead to increased treatment with moxifloxacin or a similar agent instead of azithromycin [57]. A major issue is that moxifloxacin is expensive, difficult to obtain in many parts of the world (it is not available in Brazil through the public health system [22]) and is already showing resistance to some strains [62]. The risks and benefits of screening and, above all, treating infections caused by MG and especially *Mollicutes* spp. should be carefully considered.

As for the distribution of *Mollicutes* between the anatomical sites, there was a higher prevalence of oral and anal infections than urethral infections, which may be a reason why the missed detection rate would be high if only the urethral site had been screened (67.3%). Our findings corroborate those of other studies, which have found 53–85% of chlamydial infections, 64–77.9% of gonococcal infections, and 71.4% of MG infections in extragenital sites, meaning they would not be detected and treated if only urethral screening were performed [37, 63, 64]. These results underline the importance of screening for extragenital STIs, which are often neglected due to the lack of symptoms in most of such infections, thereby implying a failure to detect and treat them, along with their increased prevalence and transmission to other sites in sexual partners [5, 41, 65, 66].

Regarding the occurrence of co-infections, 31.6% of all the participants who tested positive for some species of *Mollicutes* had two or more simultaneous infections, 92% of which involved UU. A study with MSM who attended a genitourinary clinic observed that UU and UP only ever appeared in co-infections with *Gardnerella vaginalis* or other *Mollicutes* [38]. In our study, MH also had a high prevalence in co-infections (8.5%, 21/246) and appeared more in conjunction with other bacteria than in mono-infections (4.9%, 12/246). A similar result was also found by Amorim et al. (2019) [67] in an investigation of the prevalence of STIs in people treated at specialized STI/HIV clinics in Salvador (Brazil), where MH was more prevalent as a co-infection than a mono-infection.

Species of *Mollicutes* can also co-occur with other microorganisms, such as gonorrhoea and chlamydia. Therefore, just as screening for *Mollicutes* is not recommended, screening for it only when the individual tests negative for other STIs may also be a severe problem. For instance, if patients are undiagnosed for MG, and treated for other diagnosed STIs, they may therefore be exposed to antibiotics that are resistant to or induce resistance in *Mollicutes* during such treatment [62, 68, 69]. Thus, more research is needed to assess *Mollicutes* co-infection with other microorganisms and the clinical significance of these infections, including pathogenicity at extragenital sites. More assertive screening and treatment guidelines can be developed, reducing the risks of unnecessary routine screening and inappropriate treatment.

Our results indicate a strong tendency for species of *Mollicutes* to be sexually transmitted, since variables of sexual behaviour, such as group sex and receptive anal sex, were associated with infection by *Mollicutes*, *Mycoplasma spp.*, and MH. Group sex has already been identified as a risk factor for the acquisition of STIs [70], as has receptive anal sex without the use of condoms, which is also considered a risk factor for HIV, since the mucosa in this region has many blood vessels that are easily ruptured during intercourse, thereby increasing the risk of transmission of the virus [71].

The association of these behaviours with the detection of *Mollicutes* may indicate their sexual transmission. MG has been considered an important agent of STIs for over ten years now [72–74]. The transmission of MH follows the same patterns observed in other sexually transmitted organisms, suggesting that it also occurs during condomless sex [75]. Our results, therefore, suggest that species of *Mycoplasma* and *Ureaplasma* that colonize the urogenital tract may be possible agents of STIs, which is also confirmed by the high bacterial load of MG in the anal swabs and UU in the urethral swabs taken for this study, indicating that these microorganisms may not just colonize these anatomical sites, but may also evolve to infections inducing symptoms. A study by Bissessor et al. (2016) [55] corroborates our hypothesis since the bacterial load of MG in the anal samples was higher in the MSM with proctitis than in those with asymptomatic infections (60,000 versus 10,744 copies of the organism).

As for the other variables, clinical suspicion of STI – namely, the presence of signs and/or symptoms of an STI – was also identified as a factor for the detection of *Mollicutes*. However, other microorganisms that were not investigated could be present and causing the symptoms; therefore, our results cannot be taken as evidence that the symptoms were (or were not) caused by *Mollicutes*. Thus, we emphasize the need for studies on the pathogenicity of these *Mollicutes* species in the urogenital tract, orally, and anorectally to determine to what extent and when they can be considered commensal, and when they should be regarded as etiological agents of STIs.

Given the small sample size of ATGW in our study, the statistical analysis for this subgroup could not be properly conducted. However, according to the literature, identifying as a TGW is also associated with STI acquisition [76]. Several studies show that the stigma and discrimination faced by this population make it more challenging for them to access health services [10, 20, 77–80], including specialized HIV/STI testing and prevention clinics. These barriers end up increasing the prevalence and incidence of STIs in this population [81]. In addition, trans people also face obstacles when entering the formal job market [82–84], which is again due to the stigma and discrimination they face, meaning that they often end up as sex workers as their options to make a living are very limited. To complicate matters, they often offer their services in unsafe conditions, making them vulnerable to anal sex without the use of condoms [20, 85] and sexual abuse [20]. Specifically, accessible counselling and prevention strategies are greatly needed for this population. In addition, fighting stigma and discrimination and implementing public policies that guarantee adolescent and young TGW access to education and jobs are essential to prevent such situations of vulnerability and risk.

Our study has some limitations. First of which is that our study population was based on convenience sampling. Our results may not represent the real prevalence of *Mollicutes* in the total population of adolescent MSM and TGW from Salvador, Brazil, let alone elsewhere, and particularly not in the case of TGW, given the very low sample size of this population. Also, given the overall low sample size of the study, the detection capacity of the statistical tests was limited and many results were not statistically significant. Furthermore, as the study was cross-sectional, it could not be ascertained whether any of the infections detected, symptomatic or not, were new or persistent. Also, we did not perform genotypic antibiotic resistance testing, limiting the selection of an adequate treatment strategy. Finally, the bacterial load was not quantified, limiting our ability to differentiate colonization from infection (especially in the *Ureaplasma* spp.).

## Conclusions

A high prevalence of these microorganisms was found, especially in extragenital sites, and a significant association existed between sexual behaviour and detection of *Mollicutes*. Therefore, we stress the need to raise awareness in this population regarding the importance of taking preventive measures against the transmission of *Mollicutes* during sexual intercourse, including oral sex. Further research on adolescents is required to understand their epidemiological profile in other regions and sociodemographic contexts, investigate the pathogenesis of *Mollicutes* infections in the oropharynx and rectum, and evaluate the benefits of multisite screening for these microorganisms in high-risk adolescents before implementing it in clinical practice.

## Supplementary Information

**Additional file 1: Supplementary Table 1. **Bivariate analysis of the prevalence of *M. genitalium, M. hominis*, *U. urealyticum, *and* U. parvum *among AMSM (*N*=231). PrEP1519, Salvador, Brazil, April 2019 to February 2021. **Supplementary Table 2.** Partial Socio-behavioural Questionnaire applied to participants on this study. **Figure S1.** Ct value (bacterial load) of samples that were positive for *M. genitalium* (MG), *M. hominis* (MH), *U. parvum* (UP), and *U. urealyticum* (UU) from oral, anal, and urethral swabs taken from AMSM and ATGW enrolled on the PrEP1519 study in Salvador, Brazil (*N*=246), April 2019 to February 2021.

## Data Availability

The datasets used and/or analysed during the current study are available from the corresponding author on reasonable request.

## Abbreviations

MSM: Men who have sex with men
TGW: Transgender women
MG: *Mycoplasma genitalium*
MH: *Mycoplasma hominis*
UU: *Ureaplasma urealyticum*
UP: *Ureaplasma parvum*
qPCR: Quantitative polymerase chain reaction
STI: Sexually transmitted infection
HIV: Human immunodeficiency virus
AMSM: Among adolescent men who have sex with men
ATGW: Among adolescent transgender women
PrEP: HIV pre-exposure prophylaxis
DNA: Deoxyribonucleic acid
PCR: Polymerase chain reaction
PR: Prevalence ratio
CI: Confidence interval
Ct: Cycle threshold
