# Supplementary material for: Prevalence of Mollicutes among men who have sex with men and transgender women aged 15 to 19 years in Salvador, North-eastern Brazil
Source: BMC Infect Dis. 2023 Apr 18;23:244. doi: 10.1186/s12879-023-08213-z (PMC10114492; doi:10.1186/s12879-023-08213-z)
Supplement: Supplementary file 1 — Additional file 1: Supplementary Table 1. Bivariate analysis of the prevalence of M. genitalium, M. hominis, U. urealyticum, and U. parvum among AMSM (N=231). PrEP1519, Salvador, Brazil, April 2019 to February 2021. Supplementary Table 2. Partial Socio-behavioural Questionnaire applied to participants on this study. Figure S1. Ct value (bacterial load) of samples that were positive for M. genitalium (MG), M. hominis (MH), U. parvum (UP), and U. urealyticum (UU) from oral, anal, and urethral swabs taken from AMSM and ATGW enrolled on the PrEP1519 study in Salvador, Brazil (N=246), April 2019 to February 2021. [file 12879_2023_8213_MOESM1_ESM.docx]

**Supplementary Material**

**Supplementary Table 1.** Bivariate analysis of the prevalence of *M. genitalium, M. hominis*, *U. urealyticum,* and *U. parvum* among AMSM (N=231). PrEP1519,

Salvador, Brazil, April 2019 to February 2021.

| **Variables** | *M. genitalium* | | | | *M. hominis* | | | | *U. urealyticum* | | | | *U. parvum* | | | | |
| --- | --- | --- | --- | --- | --- | --- | --- | --- | --- | --- | --- | --- | --- | --- | --- | --- | --- |
|  | **P(%)**^†^ | **PR**^‡^ | **95% CI**^§^ | **p value** | **P(%)**^†^ | **PR**^‡^ | **95% CI**^§^ | **p value** | **P(%)**^†^ | **PR**^‡^ | **95% CI**^§^ | **p value** | **P(%)**^†^ | **PR**^‡^ | **95% CI**^§^ | **p value** |  |
| **Age** |  |  |  |  |  |  |  |  |  |  |  |  |  |  |  |  |  |
| 15 to 17 yo | 3.1 | 1.00 | - | - | 18.8 | 1.00 | - | - | 28.1 | 1.00 | - | - | 6.3 | 1.00 | - | - |  |
| 18 to 19 yo | 6.0 | 1.52 | 0.69-3.86 | 0.438 | 12.6 | 1.49 | 0.66-3.36 | 0.333 | 18.6 | 0.82 | 0.80-1.83 | 0.196 | 2.5 | 0.48 | 0.50-1.32 | 0.264 |  |
| **Race/colour** |  |  |  |  |  |  |  |  |  |  |  |  |  |  |  |  |  |
| Not black | 3.0 | 1.00 | - | - | 9.1 | 1.00 | - | - | 15.2 | 1.00 | - | - | 3.0 | 1.00 | - | - |  |
| Black | 6.1 | 2.00 | 0.27-14.94 | 0.484 | 14.1 | 1.55 | 0.50-4.84 | 0.446 | 20.7 | 1.36 | 0.58-3.21 | 0.473 | 3.0 | 1.06 | 0.12-8.08 | 1.000 |  |
| **Schooling** |  |  |  |  |  |  |  |  |  |  |  |  |  |  |  |  |  |
| Elementary school/Adult education | 1.0 | 1.00 | - | - | 5.6 | 1.00 | - | - | 11.1 | 1.00 | - | - | 5.6 | 1.00 | - | - |  |
| High school/Higher education | 6.2 | 1.55 | 0.21-11.42 | 0.606 | 13.3 | 2.39 | 0.34-16.62 | 0.379 | 20.4 | 1.83 | 0.48-6.98 | 0.374 | 2.8 | 0.51 | 0.06-4.04 | 0.525 |  |
| **Sexual Orientation** |  |  |  |  |  |  |  |  |  |  |  |  |  |  |  |  |  |
| Homosexual/Gay/Lesbian | 5.1 | 1.00 | - | - | 14.1 | 1.00 | - | - | 23.1 | 1.00 | - | - | 0.6 | 1.00 | - | - |  |
| Bisexual/Heterosexual | 6.7 | 1.30 | 0.44-3.85 | 0.635 | 12.0 | 0.85 | 0.41-1.76 | 0.663 | 13.3 | 0.58 | 0.30-1.10 | 0.096 | 8.0 | **12.48** | **1.52-102.28** | **0.019** |  |
| **Steady sexual partner in the last three months** |  |  |  |  |  |  |  |  |  |  |  |  |  |  |  |  |  |
| No | 3.9 | 1.00 | - | - | 11.7 | 1.00 | - | - | 23.5 | 1.00 | - | - | 3.9 | 1.00 | - | - |  |
| Yes | 7.1 | 1.81 | 0.57-5.71 | 0.304 | 13.4 | 1.14 | 0.57-2.27 | 0.715 | 16.5 | 0.70 | 0.42-1.19 | 0.188 | 2.4 | 0.60 | 0.13-2.63 | 0.501 |  |
| **Casual sexual partner in the last three months** |  |  |  |  |  |  |  |  |  |  |  |  |  |  |  |  |  |
| No | 5.1 | 1.00 | - | - | 10.1 | 1.00 | - | - | 17.7 | 1.00 | - | - | 5.1 | 1.00 | - | - |  |
| Yes | 6.0 | 1.19 | 0.38-3.74 | 0.771 | 14.0 | 1.38 | 0.64-2.98 | 0.409 | 20.7 | 1.17 | 0.65-2.06 | 0.597 | 2.0 | 0.39 | 0.09-1.72 | 0.217 |  |
| **Receptive anal sex** |  |  |  |  |  |  |  |  |  |  |  |  |  |  |  |  |  |
| No | 3.1 | 1.00 | - | - | 6.2 | 1.00 | - | - | 15.4 | 1.00 | - | - | 4.6 | 1.00 | - | - |  |
| Yes | 6.7 | 2.18 | 0.49-9.60 | 0.284 | 15.2 | 2.48 | 0.90-6.85 | 0.081 | 21.3 | 1.39 | 0.73-2.64 | 0.318 | 2.4 | 0.53 | 0.12-2.30 | 0.396 |  |
| **Insertive anal sex** |  |  |  |  |  |  |  |  |  |  |  |  |  |  |  |  |  |
| No | 3.6 | 1.00 | - | - | 10.7 | 1.00 | - | - | 17.9 | 1.00 | - | - | 2.4 | 1.00 | - | - |  |
| Yes | 6.9 | 1.93 | 0.55-6.84 | 0.383 | 13.8 | 1.29 | 0.61-2.70 | 0.504 | 20.7 | 1.16 | 0.66-2.02 | 0.606 | 3.5 | 1.45 | 0.29-7.33 | 0.654 |  |
| **Condom use in the last three months** |  |  |  |  |  |  |  |  |  |  |  |  |  |  |  |  |  |
| Consistent | 2.2 | 1.00 | - | - | 12.2 | 1.00 | - | - | 18.9 | 1.00 | - | - | 3.3 | 1.00 | - | - |  |
| Inconsistent | 7.9 | 3.56 | 0.81-15.74 | 0.083 | 12.9 | 1.06 | 0.52-2.14 | 0.872 | 20.1 | 1.06 | 0.62-1.83 | 0.816 | 2.9 | 0.86 | 0.19-3.78 | 0.845 |  |
| **Group sex** |  |  |  |  |  |  |  |  |  |  |  |  |  |  |  |  |  |
| No | 4.7 | 1.00 | - | - | 10.0 | 1.00 | - | - | 19.5 | 1.00 | - | - | 3.2 | 1.00 | - | - |  |
| Yes | 10.3 | 2.16 | 0.70-6.69 | 0.244 | 25.6 | **2.56** | **1.29-5.09** | **0.007** | 20.5 | 1.05 | 0.53-2.09 | 0.882 | 2.5 | 0.81 | 0.10-6.59 | 0.845 |  |
| **Interference of alcohol in condom use** |  |  |  |  |  |  |  |  |  |  |  |  |  |  |  |  |  |
| No | 5.7 | 1.00 | - | - | 13.8 | 1.00 | - | - | 18.9 | 1.00 | - | - | 3.1 | 1.00 | - | - |  |
| Yes | 10.3 | 1.81 | 0.59-5.59 | 0.290 | 12.8 | 0.93 | 0.37-2.30 | 0.869 | 25.6 | 1.36 | 0.73-2.54 | 0.337 | 2.6 | 0.82 | 0.09-6.82 | 0.851 |  |
| **Interference of drugs in condom use** |  |  |  |  |  |  |  |  |  |  |  |  |  |  |  |  |  |
| No | 10.1 | 1.00 | - | - | 11.1 | 1.00 | - | - | 20.2 | 1.00 | - | - | 4.0 | 1.00 | - | - |  |
| Yes | 7.1 | 0.71 | 0.10-5.16 | 0.727 | 14.3 | 1.29 | 0.32-5.24 | 0.726 | 14.3 | 0.71 | 0.18-2.72 | 0.614 | - | **-** | **-** | **-** |  |
| **Clinical suspicion of STI** |  |  |  |  |  |  |  |  |  |  |  |  |  |  |  |  |  |
| Normal test result | 5.0 | 1.00 | - | - | 13.1 | 1.00 | - | - | 19.6 | 1.00 | - | - | 3.0 | 1.00 | - | - |  |
| Altered test result | 9.5 | 1.89 | 0.44-8.11 | 0.321 | 23.8 | 1.82 | 0.78-4.25 | 0.165 | 23.8 | 1.21 | 0.54-2.75 | 0.641 | 4.8 | 1.67 | 0.20-12.55 | 0.666 |  |

^†^P = Prevalence of the microorganism; ^‡^PR = crude prevalence ratio; ^§^95% CI: 95% confidence interval; MSM = men who have sex with men; TGW = transgender women; STI = sexually transmitted infection. Statistically significant variable (p<0.05) marked in bold.

**Supplementary Table 2.** Partial Socio-behavioural Questionnaire applied to participants on this study.

| **Questions** | **Answers** | **Variable** | **Categorization** |
| --- | --- | --- | --- |
| N.A. | N.A | **Age** | **15-17 y/o**  **18-19 y/o** |
| N.A | 1. Black 2. Brown 3. Yellow 4. White 5. Indigenous | **Race/colour** | **Black**  (Answers 0 – 1)  **Non-Black**  (Answers 2 – 4) |
| What school grade are you attending (or what was the last one you attended)? | 1. 1st year of elementary school 2. 2nd year of Elementary School 3. 3rd year of Elementary School 4. 4th year of Elementary School 5. 5th year of Elementary School 6. 6th year of Elementary School 7. 7th year of Elementary School 8. 8th year of Elementary School 9. 9th grade of elementary school 10. 1st year of high school 11. 2nd year of high school 12. 3rd year of high school 13. Higher Education 14. EJA – Youth and Adult Education and other supplements 15. I didn't go to school   15. I don't want to answer | **Schooling** | **Elementary school/Adult education** (Answers 0 – 8, 13-14)  **High school/Higher education**  (Answers 9 – 12) |
| Do you consider yourself: | 1. Cis man 2. Transgender woman 3. Transvestite 4. I don't know / I don't want to answer 5. Other. Specify: | **Population Group** | **AMSM**  (Answer 0)  **ATGW**  (Answers 1 – 4) |
| Which of these sexual orientations do you most identify with? | 1. Homosexual 2. Gay 3. Lesbian 4. Bisexual 5. Heterosexual 6. Other. Please specify: 7. I don't know/I don't want to answer | **Sexual Orientation** | **Homosexual/Gay/Lesbian**  (Answers 0 – 2, 5-6)  **Bisexual/Heterosexual**  (Answers 3 – 4) |
| In the last 3 months, have you had any sexual partner that you consider steady? | 1. No 2. Yes | **Steady Sexual Partner in the last three months** | **No**  **Yes** |
| In the past 3 months, have you had casual sexual partners? | 1. No 2. Yes | **Casual sexual partner in the last three months** | **No**  **Yes** |
| How often have you had receptive anal sex with your steady partner(s) in the past 3 months? | 1. Never 2. Rarely 3. Sometimes 4. Often 5. Always 6. I don't want to answer | **Receptive anal sex** | **No**  (Answer 0, for both questions)  **Yes**  (Answers 1 – 4, for one or both questions) |
| How often have you had receptive anal sex with your casual partner(s) in the past 3 months? |  |  |  |
| How often have you had insertive anal sex with your steady partner(s) in the past 3 months? | 1. Never 2. Rarely 3. Sometimes 4. Often 5. Always 6. I don't want to answer | **Insertive anal sex** | **No**  (Answer 0, for both questions)  **Yes**  (Answers 1 – 4, for one or both questions) |
| How often have you had insertive anal sex with your casual partner(s) in the past 3 months? |  |  |  |
| In these receptive relationships, did your partner(s) use a condom? | 1. Never 2. Rarely 3. Sometimes 4. Often 5. Always 6. I don't want to answer | **Condom use in the last three months** | **Consistent**  (Answer 4 for both questions)  **Inconsistent**  (Answers 0 – 3, for one or both questions) |
| In these insertive relationships, did you use a condom? |  |  |  |
| In the past 3 months, have you had group sex, i.e. with two or more people at the same time? | 1. Yes, once 2. Yes, more than once 3. No, not once 4. I don’t want to answer | **Group sex** | **No**  (Answer 2)  **Yes**  (Answers 0 – 1) |
| In the past 3 months, how often would you say that alcohol interfered with using condoms during sex? | 1. Never 2. Rarely 3. Sometimes 4. Often 5. Always 6. I don't know 7. I don’t want to answer | **Interference of alcohol in condom use** | **No**  (Answer 0)  **Yes**  (Answers 1 – 4) |
| In the past 3 months, how often would you say that the effects of drugs made it difficult for you to use a condom during your sexual intercourse? | 1. Never 2. Rarely 3. Sometimes 4. Often 5. Always 6. I don’t know 7. I don’t want to answer | **Interference of drugs in condom use** | **No**  (Answer 0)  **Yes**  (Answers 1 – 4) |

N.A.= Non-applicable; y/o= years old.

**Figure S1.** Ct value (bacterial load) of samples that were positive for *M. genitalium* (MG), *M. hominis* (MH), *U. parvum* (UP), and *U. urealyticum* (UU) from oral, anal, and urethral swabs taken from AMSM and ATGW enrolled on the PrEP1519 study in Salvador, Brazil (N=246), April 2019 to February 2021.
